# Machining Stresses and Initial Geometry on Bulk Residual Stresses Characterization by On-Machine Layer Removal

**DOI:** 10.3390/ma13061445

**Published:** 2020-03-22

**Authors:** Maria Aurrekoetxea, Luis Norberto López de Lacalle, Iñigo Llanos

**Affiliations:** 1 Manufacturing Processes Group, IDEKO, 20870 Elgoibar, Spain; illanos@ideko.es; 2 Department of Mechanical Engineering, Bilbao Faculty of Engineering, UPV-EHU, 48013 Bilbao, Spain; norberto.lzlacalle@ehu.eus

**Keywords:** machining distortion, analytical modeling, layer removal, Al7050 T7451 alloy, multifactor coupling

## Abstract

Prediction and control of machining distortion is a primary concern when manufacturing monolithic components due to the high scrap and rework costs involved. Bulk residual stresses, which vary from blank to blank, are a major factor of machining distortion. Thus, a bulk stress characterization is essential to reduce manufacturing costs linked to machining distortion. This paper proposes a method for bulk stress characterization on aluminium machining blanks, suitable for industrial application given its low requirements on equipment, labour expertise, and computation time. The method couples the effects of bulk residual stresses, machining stresses resulting from cutting loads on the surface and raw geometry of the blanks, and presents no size limitations. Experimental results confirm the capability of the proposed method to measure bulk residual stresses effectively and its practicality for industrial implementation.

## 1. Introduction

Machining distortion (MD) remains a costly issue within the aerospace industry, especially for monolithic and thin-walled parts, for which up to 90% of the material is removed [1]. Indeed, a great effort is still put into solving this recurring problem. In fact, the final distortion comes from a combination of different sources such as bulk or blank-initial residual stresses (BIRS), machining-induced residual stresses (MIRS), thermal deformation due to heat generation during machining, and deformation caused by clamping and other process forces, as it was reported by Brinksmeier et al. [2].

In the case of aluminium alloys, some authors [3,4,5,6] reported that the main cause for MD lies in the redistribution of the BIRS profile after the material is removed and a new equilibrium is reached. Even in cases at which machining blanks are stress relieved treated, BIRS are still a major source of distortion due to the high asymmetry of aerospace monolithic components with thin walls and webs.

However, measuring accurately BIRS all along the blank volume is not an easy task. Different techniques were developed to tackle this challenge, namely, the crack compliance method [7,8], neutron diffraction method as Bilkhu et al. [9], and also Robinson et al. [10] defined the contour method by Zhang [11] and Hill and Olson [12], or layer removal techniques proposed by Treuting in 1951 [13], or Schajer and Prime [14]. Despite being the methods currently employed, most of them are limited to the laboratory environment because of the expensive equipment requirements and the need of specially trained workers to be implemented. These facts, together with the variability of BIRS due to subtle deviations on composition or on the upstream thermomechanical processing of workpiece material supply, make it unfeasible to anticipate the actual BIRS of the part. Hence, Yang et al. [15] analyzed the fluctuations of Al 7050-T4751 BIRS amplitude and its effects on the distortion of single-sided stringer parts. Hence, defining machining processes that would yield a minimum or acceptable part distortion is a challenging task.

In order to face this problem, some authors proposed modified techniques for measuring BIRS based on the traditional methods and show potential to overcome some of the limitations aforementioned. Amongst them, on-machine layer removal can be highlighted [16,17,18], due to its potential for industrial implementation. The traditional layer-removal method is time-consuming as it requires strain gauges to be repeatedly glued and removed. This modified method enables the identification of the two-dimensional BIRS profile and can be carried out in a standard machine tool by untrained staff on actual size parts. The measuring task is performed with a standard touch probe and can be fully automated. In comparison with other techniques, which are restricted in size to laboratory test specimens, BIRS profiles of much larger components can be determined.

Furthermore, the modified formulation, makes it possible to leave the ribs unmachined [16]. Thereby, it becomes a quasi-non-destructive measuring technique, thus, the characterized blanks are still usable to machine the final component geometry. BIRS profiles obtained by the modified layer removal method show good agreement with the ones measured by other methods and published in literature [7,8,19], especially considering the variability of the stress amplitude between different machining blanks.

Once stress profiles are known, distortion minimization is achieved by simulating the material removal process using numerical, analytical, or empirical calculation models. Different approaches can be found in bibliography, which assume certain inputs to be the main cause or most critical. One model for predicting MD on complex thin wall structural parts considered the effects of mechanical load and thermal load induced by the machining process [20]. Another machining deformation prediction FE model [21] was developed considering multifactor coupling effects including BIRS, clamping loads, milling mechanical loads, milling thermal loads, and MIRS. This showed that BIRS led to the bending deformation of the whole workpiece, while MIRS of the rib or web caused a net twisting deformation. One investigation in Al 7050-T7451 revealed that BIRS are the main effect of MD of thin-walled plates [22]. On the contrary, under different processing conditions the same material experienced, below a critical depth, that MIRS were the primary contributor [23]. A study using a numerical model for MD calculation found that the main sources of error of the prediction came from inaccuracy when measuring BIRS due to its difficulty [24]. In fact, even though several calculation models are based on FE simulation, analytical models are more attractive for industrial implementation from a computational time and CPU effort perspective. In this regard, a work based on an analytical model predicted frame part deformation in 1D considering MIRS and BIRS, in which BIRS were determined using the slope method [25]. Following a similar formulation, an analytical model developed for machining deformation prediction established quantitative relationships between deformation and biaxial BIRS under three typical machining strategies, as Gao et al. reported [26]. Both analytical models were verified by FE simulations and experimental test.

The premise for minimizing the distortion of machined components goes through mastering the accuracy on BIRS profiles characterization. The present work introduces an upgraded formulation of the modified layer removal method for two-dimensional BIRS characterization. This new approach considers multiple factors such as BIRS, MIRS, and raw geometry (initial deformation) of the machining blanks, increasing its accuracy and widening the applicability of the method. Then, the accuracy of the measured BIRS is evaluated simulating a full-layer machining process with an analytical model. Modeling results are compared with the experimental measurements of workpiece distortion. This is done with the traditional formulation and the formulation that couples BIRS, MIRS, and initial deformation. The present method will work as groundwork for the development of a general tool that will allow defining a machining strategy that minimizes the MD, as well as generates competitive machining times for monolithic and thin walled components.

## 2. Materials and Methods 

### 2.1. Analytical Formulation

Most volumetric stress characterization methods are based on the measurement of deformations, displacement, or strains in the remaining material, once one section is removed. In fact, removing the material from a sample also removes BIRS contained therein and, in order to achieve a new equilibrium, the geometry changes. The strategy for performing this material removal distinguishes the methods, e.g., crack compliance, contour, slope, and hole drilling. Hereinafter, a technique for on-machine BIRS measurement, based on the layer removal method is presented.

**Figure 1:**
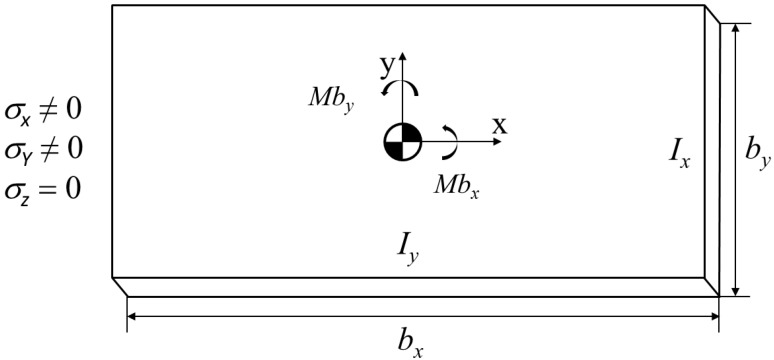
Hypothesis for bulk or blank-initial residual stresses (BIRS) characterization by the on-machine layer removal method.

In the layer removal method, material is removed layer by layer. Thus, it is suited to a flat plate and cylindrical specimens where the BIRS are known to vary in depth, but to be uniform parallel to the surface. In this specific case, plane plates are targeted for representing the typical geometry of the machining blanks for monolithic aerospace components manufacturing. These plates have of constant thickness (*H*) and width (*b_x_*, *b_y_*) in the (X) and (Y) directions, respectively (Figure 1). From the plate bending theory [27], the bending moments acting in both spatial directions (X-Y) of the plane are associated with the measured curvature in each direction. The stresses in depth (Z) direction are not considered for being negligible in comparison to the ones of the plane directions and assumed uniformly distributed along the length and width, changing only in (Z) direction (Figure 1). Furthermore, the blank material is considered isotropic and homogeneous. 

As mentioned before, BIRS values are obtained from measurements of the resulting deformations when cutting away layers of stressed material. The assessment of the unknown BIRS must be done through the inverse solution of the governing equation system represented in Equation (1), where matrix G accounts for the coefficients of the geometric and material properties of each layer, vector σ corresponds to the stresses within each of the increments, and vector d describes the set of curvatures measurements made after each layer of material is removed.

(1) G·σ=d

**Figure 2:**
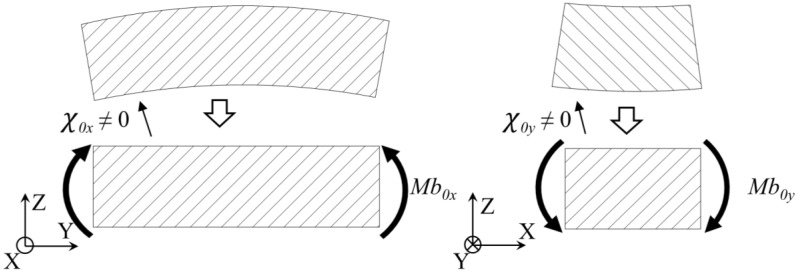
Initial deformation of the machining blank in the unclamped position and bending moment applied by the clamping.

In order to perform the machining of the layers, the workpiece is clamped to the machine-tool table, which is assumed totally flat. This way, the flatness of the raw specimen during the process is ensured. In contrast to other works [16,17,18,25,28], the raw specimen is not presumed totally flat when no material is removed (Figure 2). Thus, the clamping force is responsible for flattening the specimen, enabling the removal of constant thickness layers. Hence, the effect of the clamping force and the initial deformation is not neglected. 

**Figure 3:**
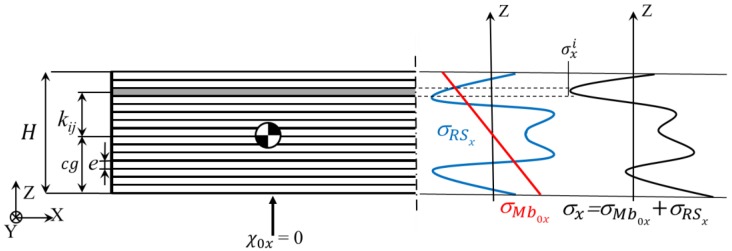
Discretization of the section in n layers of thickness e and stress acting in the cross section X when the raw part is clamped.

The bending moment of the clamping is determined from the measured initial deformation of the unclamped specimen. Its effect can be added to the formulation by introducing the stress term $\sigma_{Mb_{0}}^{i}$, related to the bending moment of the clamping force corresponding to each layer (*i*). This way, the typically symmetric stress profile of rolled machining blanks turns aside, losing its symmetry as depicted in Figure 3.

(2) $$Mb_{0x}=\frac{E\cdot I_{0x}}{\left(1-v^{2}\right)}\cdot \left(\chi_{0x}+v\cdot \chi_{0y}\right)$$

(3) $$Mb_{0y}=\frac{E\cdot I_{0y}}{\left(1-v^{2}\right)}\cdot \left(\chi_{0y}+v\cdot \chi_{0x}\right)$$

(4) $$\sigma_{Mb_{0x}}^{i}=\frac{Mb_{0x}\cdot k_{ij}}{I_{0x}}=\frac{E\cdot k_{ij}}{\left(1-v^{2}\right)}\cdot \left(\chi_{0x}+v\cdot \chi_{0y}\right)$$

(5) $$\sigma_{Mb_{0y}}^{i}=\frac{Mb_{0y}\cdot k_{ij}}{I_{0y}}=\frac{E\cdot k_{ij}}{\left(1-v^{2}\right)}\cdot \left(\chi_{0y}+v\cdot \chi_{0x}\right)$$

The blank is discretized in n horizontal layers of thickness e, which corresponds to the layer thickness machined at every step of the layer removal process (Figure 3). Naming $k_{ij}$ the distance of the center of gravity of each of the layers (i) to the center of gravity of the whole part for each process step (j) after a layer is removed. The elasticity modulus for the bulk material is represented by E, v its Poisson’s ratio, and $I_{0}$ the inertia of the cross section for each plane direction X and Y. 

This way, the total stress acting in each discretized layer $\sigma^{i}$ is the sum of the BIRS $\sigma_{RS}{}^{i}$ and the stress introduced by the clamping to straighten the curved plate $\sigma_{Mb_{0}}^{i}$. The bending moment acting in the machining blank $Mb^{RS}$ stands as the sum of the bending moment generated by each layer based on the total and averaged stress value $\sigma_{x}^{i}$, within the area $A_{i}$ and the leverage $k_{ij}$ of each layer to the neutral fiber.

(6) $$\sigma_{x}=\sigma_{Mb}{}_{0x}+\sigma_{RS}{}_{x}$$

(7) $$\sigma_{y}=\sigma_{Mb}{}_{0y}+\sigma_{RS}{}_{y}$$

(8) $$Mb_{x}^{RS}=\overset{n}{\underset{1}{\sum }}(\sigma_{x}^{i}\cdot b_{y}^{i}\cdot e\cdot k_{ij})$$

(9) $$Mb_{y}^{RS}=\overset{n}{\underset{1}{\sum }}(\sigma_{y}^{i}\cdot b_{x}^{i}\cdot e\cdot k_{ij})$$

**Figure 4:**
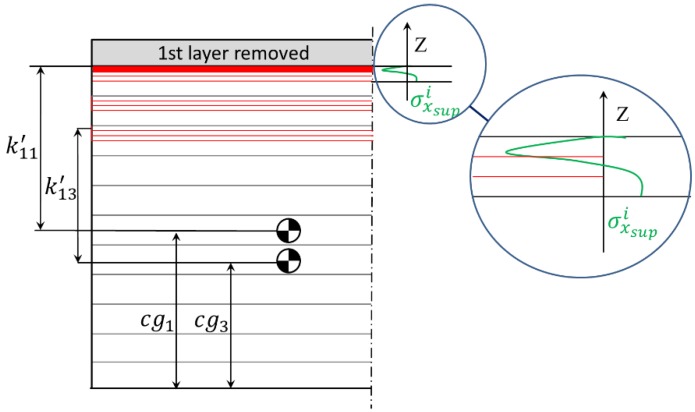
Machining-induced residual stresses (MIRS) on the surface, sublayers after machining the three first layers, and distances to the neutral plane of each of the affected layers.

Unlike the clamping effect, the machining stress values cannot be directly summed to volumetric residual stresses due to the very different depths at which they act, as they penetrate typically up to 0.2 mm depth from the surface [21,22]. The effect of MIRS is introduced similarly as with BIRS, by discretizing the depth affected by the thermomechanical loads during the machining process, being ${k_{ij}}^{\prime }$ the distance of the center of gravity of each of the sublayers (i) to the center of gravity of the whole part cg for each process step (j) after a layer is removed. The mean value of MIRS is considered uniform at each sublayer and the total effect of machining surface stresses is added to the bending moment of the plate once for each process step (j) (Figure 4).

MIRS magnitude is usually much higher than BIRS, and when the inertia of the section decreases below a critical point, as in thin-walled components, their effect can even become the primary contributor for the final machining distortion [11,23,29]. In order to introduce the effect of the MIRS at the surface in the formulation, the bending moments can be calculated similarly through Equations (10) and (11), where the distance from each sublayer to the neutral plane of the cross section is represented by $k_{ij}^{\prime }$; the average MIRS in each sublayer is represented by $\sigma_{sup}^{i}$, and $e_{s}$ is the thickness of each sublayer.

(10) $$Mb_{x}^{sup}=\overset{s}{\underset{i}{\sum }}\left(\sigma_{x_{sup}}^{i}\cdot b_{y}^{i}\cdot e_{s}\cdot k_{ij}^{\prime }\right)$$

(11) $$Mb_{y}^{sup}=\overset{s}{\underset{i}{\sum }}\left(\sigma_{y_{sup}}^{i}\cdot b_{x}^{i}\cdot e_{s}\cdot k_{ij}^{\prime }\right)$$

The total bending moment acting in the cross section in X and Y directions ($Mb_{x}$, $Mb_{y}$) is calculated as the sum of the different bending moments contribution and related with their specific plate curvature values ($\chi_{x}$, $\chi_{y}$) for a finite plate.

(12) $$Mb_{x}=Mb_{x}^{RS}+Mb_{0x}+Mb_{x}^{sup}=\frac{E\cdot I_{x}}{\left(1-v^{2}\right)}\cdot \left(\chi_{x}+v\cdot \chi_{y}\right)$$

(13) $$Mb_{y}=Mb_{y}^{RS}+Mb_{0y}+Mb_{y}^{sup}=\frac{E\cdot I_{y}}{\left(1-v^{2}\right)}\cdot \left(\chi_{y}+v\cdot \chi_{x}\right)$$

Combining the equation system represented in Equation (1) and the total bending moment acting in the machining blank, stresses can be obtained through Equation (14), where $G_{nm}$ are the products of each layer area multiplied by its leverage to the neutral fiber; $Mb_{0}$ are the bending moments due to the initial deformation; and $Mb_{m}^{sup}$ are the bending moments due to MIRS. For the first layer the effect of machining stresses cannot be added ($Mb_{x1}^{sup}=0)$, as the upper surface of this layer is unmachined and therefore stresses are not induced. The parameters for the Y direction are defined in the same way.

(14) $$\left[\begin{array}{cccc} -b_{1}\cdot e_{1}\cdot k_{11} & 0 & \cdots & 0\\ -b_{1}\cdot e_{1}\cdot k_{21} & -b_{2}\cdot e_{2}\cdot k_{22} & \cdots & 0\\ \vdots & \vdots & \ddots & \vdots \\ -b_{1}\cdot e_{1}\cdot k_{m1} & -b_{2}\cdot e_{2}\cdot k_{m2} & \cdots & -b_{n}\cdot e_{n}\cdot k_{mn} \end{array}\right]\cdot \left\{\begin{array}{c} \sigma_{x}^{1}\\ \sigma_{x}^{2}\\ \vdots \\ \sigma_{x}^{n} \end{array}\right\}=\left\{\begin{array}{c} \frac{(\chi_{x}^{1}+\nu \cdot \chi_{y}^{1})\cdot E\cdot I_{x1}}{\left(1-v^{2}\right)}-Mb_{0x}\\ \frac{(\chi_{x}^{2}+\nu \cdot \chi_{y}^{2})\cdot E\cdot I_{x2}}{\left(1-v^{2}\right)}-Mb_{0x}+Mb_{x2}^{sup}\\ \vdots \\ \frac{(\chi_{x}^{m}+\nu \cdot \chi_{y}^{m})\cdot E\cdot I_{xm}}{\left(1-v^{2}\right)}-Mb_{0x}+Mb_{xm}^{sup} \end{array}\right\}$$

The stress profile obtained corresponds to the sum of BIRS and stresses introduced by the clamping due to the initial deformation. Hence, the stresses within the machining blank can be from this point easily obtained through Equations (6) and (7).

### 2.2. Experimental Implementation

The method herein introduced was used for measuring BIRS in three machining plates of aluminium alloy Al7050-T7451, material that is common for aeronautical structural components due to its mechanical properties (Table 1). Starting from a rolled and prestretched plate size of 200 mm × 400 mm × 31.5 mm, successive layers of a thickness (e) 1 mm were machined leaving a 3 mm unmachined thickness. Due to the expected symmetry of the stress profile typical of rolled materials, the thickness machined at the central layer was higher than the rest (1.5 mm).

As mentioned, in the on-machine layer removal method both machining and measuring operations are carried out with a conventional milling machine, milling tools, and a touch probe. The tests were performed on a Soraluce™ FMT 4000 multitasking machine. The face milling was carried out using a Ø 40 mm milling tool with three Ceratizit™ XDHX 190404 FR-27P square milling type inserts with a round corner of 5 mm and clearance angle of 15°. The cutting conditions employed during the machining process can be seen in Table 2.

**Figure 5:**
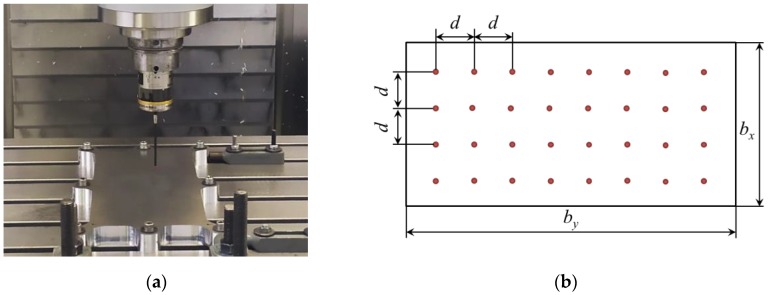
Distortion measurement with touch-probe: On-machine measurements (**a**), and touchpoint grid (**b**).

A machining-measuring sequence was repeated m times, so information related to different depths of the plate could be obtained. After each layer was removed, the specimen was unclamped and the distortion generated on it was measured automatically as the displacement in (Z) direction using the Renishaw™ RMP600 high-accuracy touch probe, which is depicted in Figure 5. A grid of 8 × 4 points was measured with a distance (d) of 40 mm. 

The curvature $\chi_{x}^{j}$, $\chi_{y}^{j}$ was obtained fitting these measurements for each (j) layer by least-squares regression to a quadratic surface function through Equations (15)–(17). The torque used in all clamping points and (j) sequences was measured and limited to 35 Nm. Furthermore, with the aim of accounting for the order of magnitude of the error, clamping repeatability was also checked and found to be below 0.005 mm.

(15) $$z\left(x,y\right)=A\cdot x^{2}+B\cdot y^{2}+C\cdot x\cdot y+D\cdot x+E\cdot y+F$$

(16) $$\chi_{x}=2\cdot A$$

(17) $$\chi_{y}=2\cdot B$$

To account for the effect of raw geometry or initial deformation of the machining blank, the plate in the unclamped position was measured with the touch probe in the same way as in the layer removal method. To remove the error of the surface irregularities, the unmachined clamped position was also measured and, from the difference in (Z) positions, the initial curvature was obtained.

**Figure 6:**
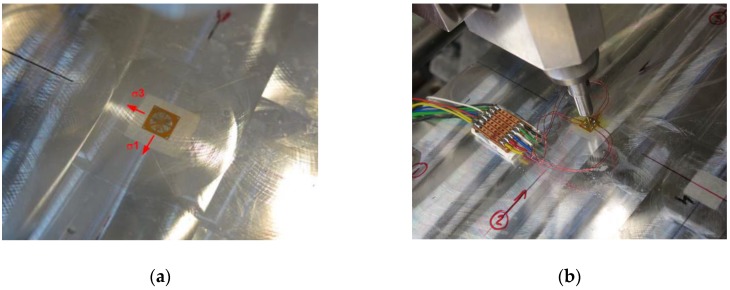
Detail of installation of strain gauge at measuring position (**a**) and incremental drilling (**b**).

In order to include the actual MIRS values generated by the cutting process, the incremental hole drilling technique (Figure 6) was used. Incremental hole drilling is a mechanical release technique for measuring near-surface stresses, which provides a measurement in the plane of the surface over a shallow region of typically 0.5–1 mm and involves drilling a small hole in the centre of a strain gage rosette. Stress is calculated based upon the measured strain change due to the drilling of the hole [30]. 

**Figure 7:**
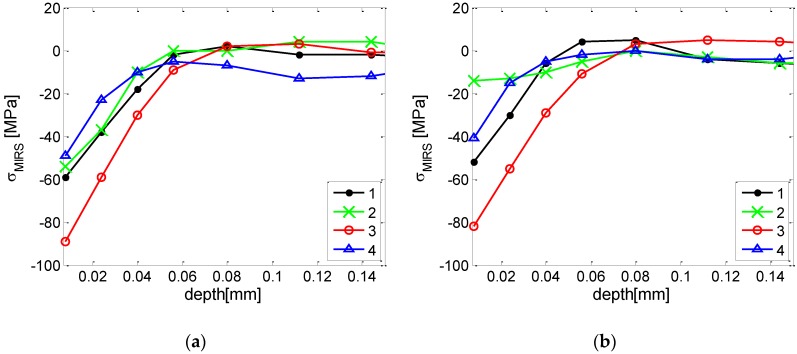
Variability of MIRS profiles measured by incremental hole drilling in four different locations: Distribution of longitudinal stresses vs. depth (**a**) and distribution of transverse stresses vs. depth (**b**).

In this case, longitudinal and transverse stresses were determined by using a target strain gauge and drilling a central hole to a maximum depth of 0.512 mm, even though typically in these cases MIRS was found to stabilize at approximately a depth of 0.1 mm. Indeed, distributions of stresses in summary confirm that for these specific machining parameters, the direct effects of the milling process did not appear to extend to depths beyond 0.08 mm (Figure 7). Results included the relaxed strains recorded at each drilling depth. Four different locations were selected for representing the variability of the stresses for the used machining conditions. Calculations for the data of all four measured locations were carried out, and location no. 3 was chosen for representing the worst condition.

While the cutting parameters, the cooling strategy, and the geometry and wear of the tool influence significantly on the residual stresses induced in the machined surfaces, the cutting conditions were kept constant and the tool wear was monitored and controlled, with the aim of keeping the machining stresses constant and verifying the validity of the method.

## 3. Results

**Figure 8:**
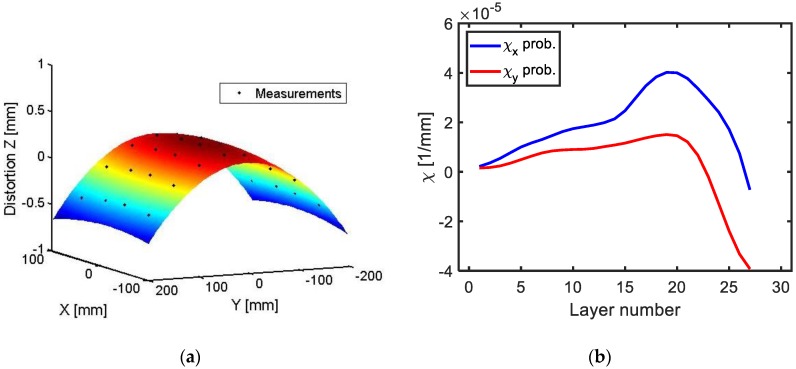
Curvature obtained from distortion measurements by quadratic regression in X and Y directions for a specific layer (**a**), and curvature evolution for all layers measured by probing in the LR test (**b**).

Following the procedure described above and performing a series of machining-measuring sequences the curvature progression was obtained as depicted in Figure 8 in X and Y directions.

The scattering on the distortion values measured with the touch probe was found to be below 3 μm and the mean coefficient of determination for the curvatures was $\bar{R^{2}}$ = 0.9789, with a standard deviation of $\sigma_{R^{2}}$ = 0.0175 for all measured (j) layers.

**Figure 9:**
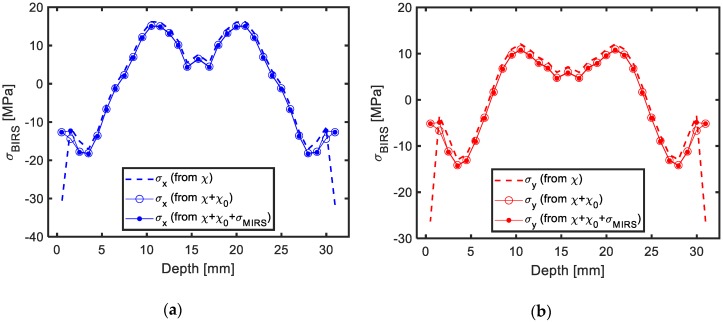
Measured BIRS profile considering only bulk or blank-initial residual stresses (BIRS), BIRS and initial deformation of the raw plate $\chi_{0}$, and BIRS, $\chi_{0}$ and machining-induced residual stresses (MIRS): X direction(**a**) and Y direction (**b**).

With curvature values, the BIRS profiles results were obtained following the proposed formulation. In Figure 9, three different BIRS profile results are shown for (X) and (Y) directions, one considering only BIRS in the formulation, another one considering BIRS and initial deformation of the raw plate $\chi_{0}$, and a final one introducing the full formulation proposed herein, BIRS, initial deformation, and MIRS. As it can be seen, the BIRS profiles results show a good agreement with the ones found in bibliography for the same material obtained by traditional methods [8,25]. 

From the results it can be determined that, while the effect of the initial deformation modifies in a certain extent the amplitude of the stresses obtained by this method, the effect of the MIRS was not as significant for the analyzed cases. Nevertheless, MIRS profiles depend on the cutting conditions and can vary remarkably if these are changed. Finding MIRS of up to 10 times higher than the ones measured is possible when high speed machining and aggressive strategies are used in this material [31] and, therefore, their effect on the measured stresses can be much higher for those cases.

## 4. Discussion

In order to evaluate the work once stress data and geometry are known, the proposed formulation can be employed to simulate the machining process and predict distortion as material is removed [16,25].

**Figure 10:**
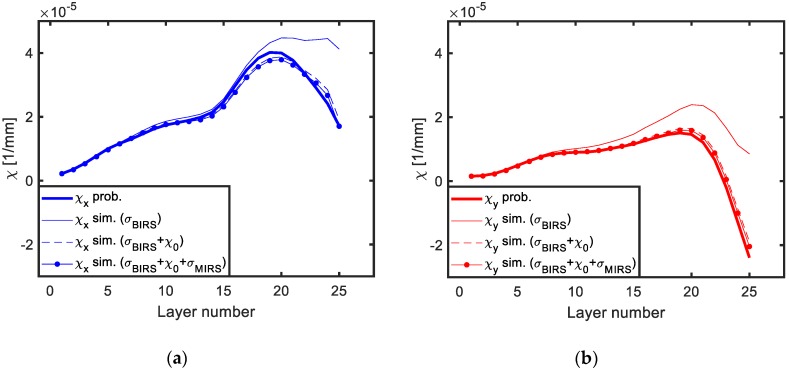
Experimental vs. simulated curvatures for the LR process of a machining plate with known BIRS profile (measured by on-machine layer removal method): X direction (**a**) and Y direction (**b**).

The plots depicted in Figure 10 show the curvature progression for all the process steps (j), as layers of the machining blank are removed. In each plot the experimental measurements can be seen in comparison to three different simulations for the same stress data input considering different effects (only BIRS; BIRS and initial deformation; and BIRS, MIRS, and initial deformation). 

As it can be seen, if only BIRS profiles are considered in the stress characterization, the predicted machining distortion presents a large error in comparison to the experimental results. However, when the effects of initial deformation and MIRS are coupled in the BIRS characterization, the simulation of the layer removal provides curvature results in good agreement with the experimental measurements.

**Figure 11:**
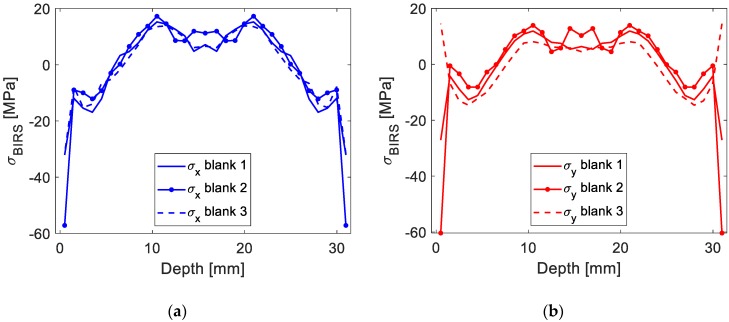
Measured BIRS profile in three Al7050-T7451 machining blanks considering only BIRS: In X direction (**a**), and in Y direction (**b**).

**Figure 12:**
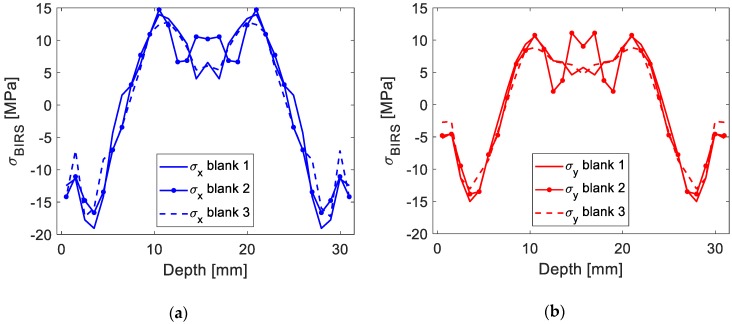
Measured BIRS profile in three Al7050-T7451 machining blanks considering BIRS, $\chi_{0}$, and MIRS: In X direction (**a**), and in Y direction (**b**).

In the figures below the results using the original formulation (Figure 11) and the coupled formulation here introduced (Figure 12) are compared for three different machining blanks. With the coupled formulation the accuracy of the method in BIRS characterization is improved, concerning both extreme stresses values for the near-surface layers and average variability. While considering only BIRS the difference between blanks reaches values up to 70 MPa, coupling BIRS, initial deformation, and MIRS the maximum stress difference is lower than 5 MPa. 

**Figure 13:**
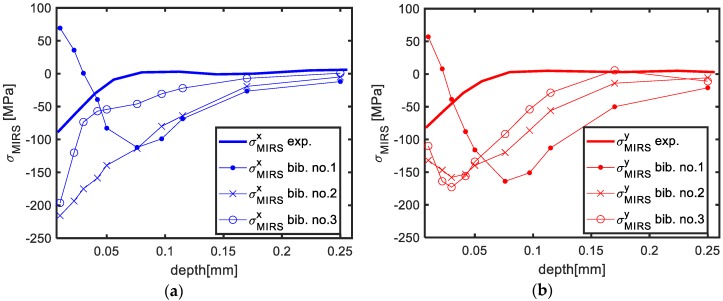
MIRS profiles in Al7050-T7451 blanks for different machining parameters: Experimental vs. results from bibliography [35] in X direction (**a**), and in Y direction (**b**).

Finally, a parametric sensitivity analysis of the bulk residual stress characterization method to different machining parameters is presented. Introducing different MIRS profiles from bibliography [32,33,34,35] to the proposed formulation, the effect of variations of the machining parameters is assessed. Amongst all the data evaluated, three MIRS profiles obtained by Perez et al. [35] are chosen, due to their greater penetration depth (Figure 13) in comparison to other examined cases. In Table 3, the machining parameters corresponding to each MIRS profile are described.

**Figure 14:**
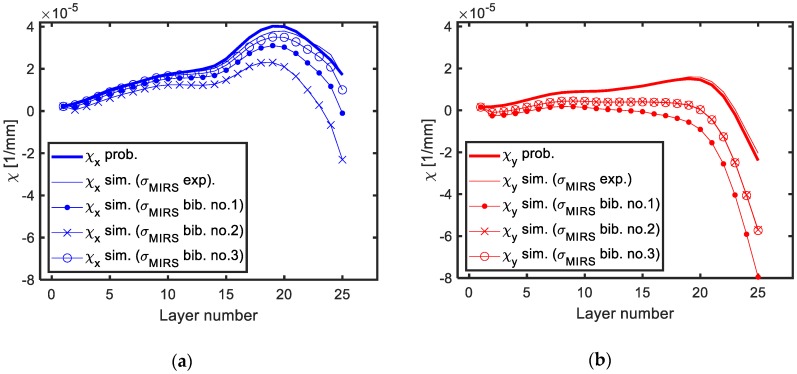
Sensitivity analysis of the LR method to different machining parameters: Actual curvatures measured by probing, simulation results from experimental MIRS, and simulation results from bibliography MIRS [35] in X direction (**a**) and in Y direction (**b**).

As shown in Figure 14, the predicted machining distortion using the LR method presents a large error when MIRS which do not correspond to the prescribed machining scenario are introduced. This is because on-machine measurements only reflect the effect of actual MIRS generated during the process. Variations in amplitude or penetration of surface stresses, such as the MIRS from bibliography introduced, affect significantly the accuracy of the BIRS obtained by the on-machine LR method. Thus, the accurate identification of the MIRS generated on the machined surfaces during LR tests is mandatory in order to obtain accurate results for the BIRS.

## 5. Conclusions

The present work proposes a method for bulk residual stresses (BIRS) characterization integrating different aspects. The importance of coupling these main effects acting on the machining plates in order to measure BIRS accurately using the on-machine method is demonstrated. After introducing the formulation, the experimental tests procedure is presented and results discussed obtain the following conclusions:

- The effects of the initial deformation and machining stresses (MIRS) must be considered for BIRS characterization due to the big influence in stress values, especially near the surface.
- Using the formulation to perform inversely the calculation of distortion, a check of the results accuracy can be done, as well as a prediction of machining distortion for face milling operations with a negligible computation time.
- The proposed characterization method enables the identification of the BIRS from measured curvatures on machining blanks accurately.
- Parametric sensitivity analysis confirms that accurate assessment of MIRS is necessary to obtain reliable results of bulk residual stresses (BIRS) by the on-machine LR method.
- The effect of the different machining conditions on the final part distortion, and by extension the effect of the MIRS, was verified by the parametric sensitivity analysis.
- The on-machine layer removal method can be implemented in industrial environments by untrained staff using common industrial machines and tools. This fact enables BIRS identification in the shop-floor and allows automation in a future prospect.

Further research will cover a sensitivity analysis to identify the significance of different cutting parameters and their corresponding relationship to the accuracy of each approach. Furthermore, the described formulation will be upgraded for its use on ribbed geometries, and therefore allow the identification of the bulk residual stresses on machining blanks, which can still be usable to machine the final components.

## Figures and Tables

**Table 1:** Mechanical properties of aluminium alloy Al7050-T7451 [23,26].

Mechanical Properties	Al7050-T7451
Tensile Yield Strength [MPa]	469
Modulus of Elasticity [GPa]	71.7
Poisson’s ratio [-]	0.33
Density [g/cm^3^]	2.83

**Table 2:** Cutting conditions employed during the experimental test.

Parameter	Value
Cutting speed—*V*c (m·min^−1^)	500
Feed per tooth—*f* (mm)	0.1
Axial depth of cut—*a_p_* (mm)	1
Radial depth of cut—*a_e_* (mm)	30

**Table 3:** Cutting parameters of MIRS measured in Al7050-T7451 face milled surfaces by incremental hole drilling.

Parameter	Exp.	Bib. No. 1	Bib. No. 2	Bib. No. 3
Tool Diameter	40	32	32	32
Cutting speed—*V*c (m·min^−1^)	500	200	800	1400
Feed per tooth—*f* (mm)	0.1	0.2	0.2	0.2
Axial depth of cut—*a_p_* (mm)	1	1	1	1
Radial engagement—(%)	75	75	75	75
Rake angle—(°)	20	11	11	11
Clearance angle—(°)	15	8	8	8
Corner radius—(mm)	0.4	0.2	0.2	0.2

## References

[1] Bowden D.M., Halley J.E. (2001). Aluminium Reliability Improvement Program Final Report 60606.

[2] Brinksmeier E., Cammett J.T., König W., Leskovar P., Peters J., Tönshoff H.K. (1982). Residual Stresses–Measurement and Causes in Machining Processes. CIRP Ann..

[3] Li J., Wang S. (2017). Distortion caused by residual stresses in machining aeronautical aluminum alloy parts: Recent advances. Int. J. Adv. Manuf. Technol..

[4] Yang Y., Li M., Li K.R. (2014). Comparison and analysis of main effect elements of machining distortion for aluminum alloy and titanium alloy aircraft monolithic component. Int. J. Adv. Manuf. Technol..

[5] Dong H., Ke Y. (2006). Study on Machining Deformation of Aircraft Monolithic Component by FEM and Experiment. Chin. J. Aeronaut..

[6] Wang Z., Chen W., Zhang Y., Chen Z., Liu Q. (2005). Study on the Machining Distortion of Thin-walled Part Caused by Redistribution of Residual Stress. Chin. J. Aeronaut..

[7] Nervi S., Szabó B.A. (2007). On the estimation of residual stresses by the crack compliance method. Comput. Methods Appl. Mech. Eng..

[8] Prime M.B., Hill M.R. (2002). Residual stress, stress relief, and inhomogeneity in aluminum plate. Scr. Mater..

[9] Bilkhu R., Ayvar-Soberanis S., Pinna C., McLeay T. (2019). Machining Distortion in Asymmetrical Residual Stress Profiles. Procedia CIRP.

[10] Robinson J.S., Tanner D.A., Truman C.E., Wimpory R.C. (2011). Measurement and Prediction of Machining Induced Redistribution of Residual Stress in the Aluminium Alloy 7449. Exp. Mech..

[11] Zhang Z., Li L., Yang Y., He N., Zhao W. (2014). Machining distortion minimization for the manufacturing of aeronautical structure. Int. J. Adv. Manuf. Technol..

[12] Hill M.R., Olson M.D. (2014). Repeatability of the Contour Method for Residual Stress Measurement. Exp. Mech..

[13] Treuting R.G., Read W.T. (1951). A mechanical determination of biaxial residual stress in sheet materials. J. Appl. Phys..

[14] Schajer G.S., Prime M.B. (2006). Use of Inverse Solutions for Residual Stress Measurements. J. Eng. Mater. Technol..

[15] Yang Y., Yang Y., Li X., Li L., He N., Zhao G., Chen N., Lan H., Zhou Z. (2019). Investigation on deformation of single-sided stringer parts based on fluctuant initial residual stress. J. Mater. Process. Technol..

[16] Llanos I., Lanzagorta J.L., Beristain A. (2017). Part Distortion Modeling on Aluminum Slender Structural Components for Aeronautical Industry. Procedia CIRP.

[17] Dreier S., Denkena B. (2014). Determination of Residual Stresses in Plate Material by Layer Removal with Machine-integrated Measurement. Procedia CIRP.

[18] Gulpak M., Sölter J., Brinksmeier E. (2013). Prediction of Shape Deviations in Face Milling of Steel. Procedia CIRP.

[19] Gao H., Zhang Y., Wu Q., Li B. (2018). Investigation on influences of initial residual stress on thin-walled part machining deformation based on a semi-analytical model. J. Mater. Process. Technol..

[20] Hussain A., Lazoglu I. (2019). Distortion in milling of structural parts. CIRP Ann..

[21] Tang Z.T., Yu T., Xu L.Q., Liu Z.Q. (2013). Machining deformation prediction for frame components considering multifactor coupling effects. Int. J. Adv. Manuf. Technol..

[22] Huang X., Sun J., Li J. (2015). Finite element simulation and experimental investigation on the residual stress-related monolithic component deformation. Int. J. Adv. Manuf. Technol..

[23] Huang X., Sun J., Li J. (2015). Effect of Initial Residual Stress and Machining-Induced Residual Stress on the Deformation of Aluminium Alloy Plate. J. Mech. Eng..

[24] Ma Y., Zhang J., Yu D., Feng P., Xu C. (2019). Modeling of machining distortion for thin-walled components based on the internal stress field evolution. Int. J. Adv. Manuf. Technol..

[25] Wang Z., Sun J., Liu L., Wang R., Chen W. (2019). An analytical model to predict the machining deformation of frame parts caused by residual stress. J. Mater. Process. Technol..

[26] Gao H., Zhang Y., Wu Q., Song J. (2017). An analytical model for predicting the machining deformation of a plate blank considers biaxial initial residual stresses. Int. J. Adv. Manuf. Technol..

[27] Timoshenko S., Goodier J.N. (1982). Theory of Elasticity.

[28] Llanos I., Aurrekoetxea M., Agirre A., López de Lacalle L.N., Zelaieta O. (2019). On-machine Characterization of Bulk Residual Stresses on Machining Blanks. Procedia CIRP.

[29] López de Lacalle L.N., Lamikiz A., Muñoa J., Salgado M.A., Sánchez J.A. (2006). Improving the high-speed finishing of forming tools for advanced high-strength steels (AHSS). Int. J. of Adv. Manufac. Tech..

[30] ASTM (2008). E837–08 Standard Test Method for Determining Residual Stress by the Hole-Drilling Strain Gage Method.

[31] Ammula S.C., Guo Y.B. (2005). Surface Integrity of Al 7050-T7451 and Al 6061-T651 Induced by High Speed Milling. SAE Tech. Paper.

[32] Huang X., Sun J., Li J., Han X., Xiong Q. (2013). An Experimental Investigation of Residual Stresses in High-Speed End Milling 7050-T7451 Aluminum Alloy. Adv. Mech. Eng..

[33] Tang Z.T., Liu Z.Q., Wan Y., Ai X. (2008). Study on Residual Stresses in Milling Aluminium Alloy 7050-T7451. Advanced Design and Manufacture to Gain a Competitive Edge.

[34] Tang Z.T., Liu Q., Pan Y.Z., Wan Y., Ai X. (2009). The influence of tool flank wear on residual stresses induced by milling aluminum alloy. J. Mater. Process. Technol..

[35] Perez I., Madariaga A., Cuesta M., Garay A., Ruiz J.J., Rubio F.J., Sanchez R. (2018). Effect of cutting speed on the surface integrity of face milled 7050-T7451 aluminium workpieces. Procedia CIRP.

